# Does Alignment Technique in Medially Stabilized Total Knee Arthroplasty Affect the Patellofemoral Joint Biomechanics and Patient-reported Outcomes at 1 Year? A Prospective Registry-based Cohort Study

**DOI:** 10.1016/j.artd.2025.101750

**Published:** 2025-06-26

**Authors:** Samuel W. King, Nicolas Silvestrini, Anne Lübbeke, Hemant Pandit, Hermes H. Miozzari

**Affiliations:** a Leeds Institute of Rheumatic and Musculoskeletal Medicine, Chapel Allerton Hospital, University of Leeds, Leeds, UK; b Leeds Teaching Hospitals NHS Trust, St. James's University Hospital, Leeds, UK; c Faculty of Medicine, Division of Orthopaedic Surgery and Musculoskeletal Trauma Care, Geneva University Hospitals, University of Geneva, Geneva, Switzerland

**Keywords:** Medially stabilized, Patellar tilt, Patellofemoral joint, TKA, Kinematic alignment, Mechanical alignment

## Abstract

**Background:**

Current femoral components may produce a nonanatomical trochlea position with kinematic alignment (KA). This study compared effects of alignment on patellar tilt and patient-reported outcomes in medially stabilized total knee arthroplasty (MS-TKA).

**Methods:**

MS-TKA patients from a prospective registry-based consecutive cohort were subdivided by alignment technique and patella resurfacing status. Impact of alignment technique stratified by patellar resurfacing on 1-year patellar tilt was investigated with analysis of variance. For alignment technique impact stratified by patellar resurfacing on 1-year Western Ontario and McMaster Universities Arthritis Index (WOMAC), analysis of covariance including preoperative WOMAC pain and function scores as covariates were performed. Spearman's rank correlation was computed for patellar tilt vs WOMAC pain and function at 1 year.

**Results:**

295 MS-TKAs were included: mechanical alignment in 168 (56.9%), patella resurfaced in 137 (46.4%), mean age 68.0 years, mean body mass index 30.6 kg/m^2^. More women had nonresurfaced patellae (74.3%; *P* = .011), otherwise baseline demographics, radiological parameters, WOMAC, and lateral release rates were similar between groups. At 1 year, patellar tilt was higher for KA vs mechanical alignment (7.31 vs 5.90; *P* = .028) in both resurfaced and nonresurfaced TKA. No effect of alignment on baseline-adjusted WOMAC at 1 year was found in both resurfaced and nonresurfaced TKA. One-year patellar tilt did not correlate with WOMAC pain (r_s_ = −0.004; *P* = .954) or function (r_s_ = 0.016; *P* = .832).

**Conclusions:**

Despite greater patellar tilt at 1year with KA, this study suggests alignment technique in MS-TKA does not adversely impact patella-femoral joint in a clinically significant manner, leading to similar patient-reported outcomes regardless of patellar resurfacing status.

## Introduction

Total knee arthroplasty (TKA) is a popular and effective treatment for end-stage osteoarthritis [[1], [2], [3]]. However, 10%-25% of patients report dissatisfaction and a national survey of 7000 postoperative TKA patients found less than 10% have no or hardly any problems with their knees [4,5]. Traditional mechanical alignment (MA) aims to create a 180-degree hip-knee-ankle (HKA) angle in the coronal plane with a perpendicular tibiofemoral joint line [6,7]. Kinematic alignment (KA) provides patient-specific implant alignment [8] and has gained popularity in the recent years. It aims to align the femoral and tibial components to the 3 axes of the native knee and restore prearthritic leg alignment and joint obliquity. This approach does not require soft tissue releases and the literature suggests a more physiological gait and improved patient satisfaction [9], with similar 10-year survival rate [10] compared with MA.

Patellofemoral joint (PFJ) problems are a significant cause of dissatisfaction after TKA and account for approximately 10% of revision indications [11]. Femoral component alignment and prosthetic trochlear morphology are both key factors in the development of patellofemoral complications with or without resurfacing of the patella [12]. In the MA technique, external rotation and lateralization of the femoral implant reduces the Q angle to promote early patella engagement by maximizing lateral and proximal reach of the trochlea [13]. Meanwhile, in KA technique, the femoral component is implanted centrally on the distal femur without external rotation, thus potentially increasing the Q angle. Theoretically this increases the risk of patellar maltracking [[14], [15], [16]], a concern for some knee surgeons and a factor affecting widespread adoption of KA [15]. Some evidence suggests delayed patellar capture in KA TKA, a theoretical risk for PFJ instability and subsequent complications [17]. Indeed, almost all available TKAs are developed for the MA technique, potentially risking abnormal PFJ biomechanics when performing KA technique [18].

Separately from examination of alignment technique, the effect of patellar tilt in itself on clinical outcomes has been variably reported. Patellar tilt of more than 10 degrees has been shown to reduce Knee Society Score at 2 years postoperative in resurfaced and nonresurfaced patellae [19]. Another study found a patellar tilt of more than 5 degrees to be associated with a substantially higher chance of fair or poor postoperative results, and a third small study demonstrated lower patient-reported outcome measures (PROMs) scores in patients with a postoperative patellar tilt of over 4 degrees [19,20]. Meanwhile, other studies have found no evidence that patellar tilt adversely affects pain or range of motion (ROM) [21,22].

In the context of the effects of alignment technique, most studies investigating patellofemoral relationships between KA and MA knees are limited to cadaveric or computer-simulated studies which have inherent substantial limitations. A study of an older cruciate-retaining TKA implant by Koh et al. reported on PFJ issues and PFJ-related outcomes following TKA when comparing KA with MA techniques [23]. At 6 months, greater patellar tilt was noted in the KA group, but by 2 years this had resolved, and clinical outcomes were comparable at both time points. Another study demonstrated an increase in postoperative patellar tilt at 6 months, but with no difference in Oxford Knee Score or ROM [24]. The biomechanics of medially stabilized TKA (MS-TKA) differ from the previously studied implants, and there is no study in the available literature which compares PFJ biomechanics for this type of implant [25]. The Medacta GMK-Sphere knee (Global Medacta Knee SPHERE, Medacta International, Castel San Pietro, Switzerland) used in this study follows the principles of MS-TKA design. The lateral femoral component and lateral tibial baseplate are relatively flat to permit lateral femoral rollback, while the high anteromedial tibial baseplate provides constraint to provide a spherical “ball-and-socket” articulation, to better replicate native knee kinematics [26].

This study aims to compare 1-year outcomes in patients who had primary MS-TKA with either a MA or KA alignment, using a standardized surgical technique and the same modern implant. The following were assessed: the effects of alignment technique on patellar tilt, alignment technique on patient-reported outcome measures, and correlation of patellar tilt with patient-reported outcome measures.

## Material and methods

### Study design, setting, and participants

This study used a prospective register-based cohort from a large tertiary hospital. All patients included in this registry consented for their data to be used for research. Local institutional approval was provided for the study by the institutional review board. Patients who had a preoperative diagnosis of knee osteoarthritis undergoing elective primary TKA using GMK Sphere design at a university hospital by a single surgeon between September 2013 and October 2021 were screened for eligibility (n = 453).

**Figure 1:**
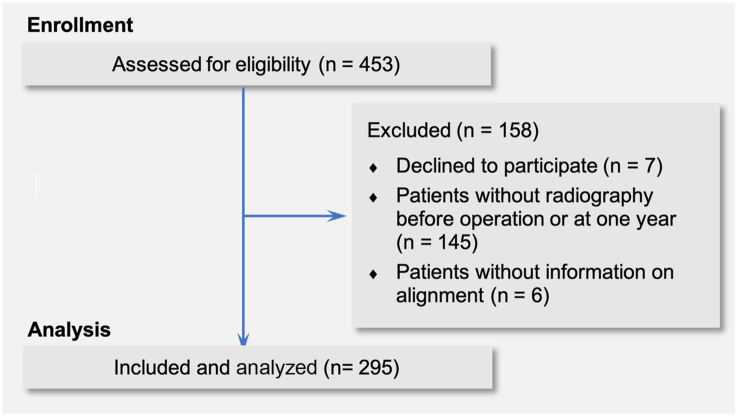
Eligibility criteria for inclusion in analysis.

Patients who had undergone a previous arthroscopy without implanted material were eligible for inclusion. Patients with both resurfaced and nonresurfaced patellae were included. Prior to 2019, the patella was rarely resurfaced throughout the institution, irrespective of the system and the constraint used. An internal review of registry data found a higher-than-expected rate of secondary patellar resurfacing across the institution. Following this, by default all patellae were resurfaced by the operating surgeon. Those who had refused consent for inclusion in the institutional arthroplasty registry or had not consented for the use of their data for research were excluded (n = 7). Patients who did not have long-leg alignment, lateral knee and Merchant skyline radiographs before operation, at 6 weeks, or 1-year postoperative were also excluded (n = 145). Of these 145 excluded on grounds of missing radiographical data, 93 (64%) had undergone MA, 45 (31%) KA, and 7 (5%) were missing data on alignment technique. Finally, patients were excluded solely for missing information on the type of alignment technique used (n = 6). Overall, 158 (34.9%) were excluded. The flowchart of the study is presented in Figure 1. The final sample included 295 TKAs. A post hoc power analysis (using the software GPower) indicates that this sample size allows detection of an effect size of 0.35 (Cohen's d) for an independent *t*-test with 80% power and 5% alpha, appropriate to investigate the impact of alignment type (MA vs KA) on patellar tilt.

### Operative technique

All patients underwent a standard medial parapatellar approach, and all components were fixed with PALACOS R + G (Heraeus Medical GmbH, Germany), an antibiotic loaded high-viscosity bone cement, mixed under vacuum as per manufacturer instructions. Intravenous tranexamic acid has been routinely used since 2013. An intra-articular suction drain was occasionally used on a case-by-case basis. A transparent absorbing waterproof wound dressing was changed at day 2 and left until removal of stitches at 2 weeks. Postoperatively, mobilization with crutches and full weight-bearing commenced on day 1 and all patients underwent a standard 6-week rehabilitation program. Deep vein thrombosis prophylaxis with low-molecular heparin was started on the day of surgery and maintained for 6 weeks.

The patella was routinely denervated and osteophytes were cleared in all cases following the arthrotomy. The influence of the medial retinaculum and the medial patellofemoral ligament was observed at opening to avoid unnecessary lateral releases. If there was concern regarding patellar tilt, patellar tracking was assessed after trialing femoral and tibial components by temporarily closing the arthrotomy with 2 sutures. Need for lateral facetectomy and/or lateral release was determined at this point, with neither being routinely performed. For resurfaced patellae, the standard GMK sphere medialized dome component was used.

MA was performed as per traditional techniques, with femoral and tibial resections perpendicular to the mechanical axes of the femur and tibia, respectively, to create a 180-degree HKA angle in the coronal plane. Unrestricted KA was performed by calipered technique, addressing the femur first and then the tibia following the technique published by Howell et al. [27], and the posterior cruciate ligament was routinely sacrificed. Both varus and valgus knees were included, with choice of alignment technique based on availability of KA equipment. Patients with bone loss or ligamentous laxity inappropriate for this system were addressed with a different knee system.

### Clinical data

Prospectively collected registry data from a local-based arthroplasty registry (GAR: Geneva Arthroplasty Registry) were analyzed. Preoperative, intraoperative, and postoperative data were extracted. Baseline preoperative data included patient age, sex, body mass index (BMI), smoking status, American Society of Anesthesiologists score, the patient-reported Western Ontario and McMaster Universities Arthritis Index (WOMAC) pain and function domains. Intraoperative data included surgical approach, type of alignment (MA vs KA), patella resurfacing status, and use of lateral release. WOMAC was again recorded at 1-year postoperative. Revision and reoperation data were collected and compared between alignment groups.

### Radiological data

Radiographs were obtained and reviewed at the following points: preoperative, 6 weeks, and at 1-year postsurgery. These included long-leg alignment, lateral knee, and Merchant skyline radiographs.

Radiographical measurements were performed by an experienced orthopaedic surgeon independent from the operating team who was blinded to the alignment technique utilized. Long leg alignment films in the coronal plane were used to measure the HKA angle to assess overall coronal knee alignment [28]. Preoperative patellar tilt was assessed in the Merchant skyline view by recording the angle between a line tangential to the most anterior aspect of the medial and femoral condyles and an equatorial line across the patella [23,29]. In nonresurfaced patellae, postoperative tilt was measured in the same way. In resurfaced patellae, postoperative patellar tilt was assessed by measuring the angle between a line tangential to the most anterior aspect of the medial and femoral condyles and a line tangential to the patellar-resurfacing bone-implant interface [29]. On the lateral knee radiograph, posterior tibial slope was measured as the angle between a line tangential to the tibial plateau and the posterior tibial cortex [30]. Patellar height was assessed using the Caton-Deschamps index in native knees [31], while derived Caton-Deschamps index was used in implanted knees [32].

### Data analyses

Patellar resurfacing was expected to affect patellar tracking. Therefore, continuous baseline variables were analyzed with 2 (resurfacing: yes or no) × 2 (alignment: mechanical or kinematic) analysis of variance (ANOVA). These included preoperative demographics (age, BMI), radiographical parameters (HKA angle, posterior tibial slope, patellar tilt, and patellar height), and PROMs (WOMAC pain and function scores). Generalized linear models were used to compare categorical preoperative demographics (sex, American Society of Anesthesiologists score, smoking status) according to a 2 (resurfacing: yes or no) × 2 (alignment: mechanical or kinematic) factorial model. These analyses revealed a higher proportion of women with patellar resurfacing compared with men. Therefore, sex was included in the subsequent analyses as a control variable.

To investigate the impact of patellar resurfacing and alignment technique on patellar tilt at 1 year, a 2 (resurfacing: yes or no) × 2 (alignment: mechanical or kinematic) × 2 (sex) ANOVA was used. To evaluate the effect of TKA on WOMAC pain and function scores at 1 year, 2 (time: baseline or 1 year) × 2 (sex) repeated-measures ANOVAs were used. To investigate the impact of patellar resurfacing and alignment technique on WOMAC pain and WOMAC function scores at 1 year, 2 (resurfacing: yes or no) × 2 (alignment: mechanical or kinematic) × 2 (sex) analysis of covariance were used including preoperative WOMAC pain and function scores as covariates, respectively.

Spearman's rank correlation was computed to assess the relationship between patellar tilt, WOMAC pain, and WOMAC function scores at 1-year postoperative.

Normality assumption was checked graphically with no major violation identified. To account for potential violation of homogeneity assumption, *P* values of parameter estimates were reported with robust standard error (Huber-White, type HC3) when appropriate. Homogeneity of regression slopes (analysis of covariance) was checked graphically with no major violation identified. Statistical significance was assessed at a 2-sided 0.05 level for all analyses. The data were analyzed using IBM SPSS Statistics (Version 28).

## Results

### Baseline and operative characteristics

Patients mean age was 68.04 years (SD = 8.79), with a mean BMI of 30.64 kg/m^2^ (SD = 6.10). Demographics, baseline radiographical and clinical data, and operative data are reported in Table 1, Table 2. The patella was resurfaced in 137 TKAs (46.4%) and nonresurfaced in 158 TKAs (53.6%). With respect to alignment technique, MA was used in 168 TKAs (56.9%) and KA in 127 (43.1%). Demographics were similar between the 4 groups except for a higher proportion of women with nonresurfaced compared to resurfaced patella (74.3% vs 59.8%; *P* = .011), in absence of other effects. Groups were also similar regarding baseline radiographical and clinical data (HKA angle, posterior tibial slope, patellar tilt, patellar height, WOMAC pain, and WOMAC function). The need for lateral release did not differ between groups.

**Table 1:** Demographic data for each of the subgroups of patients.

Characteristic	Nonresurfaced		Resurfaced		*P* values		
	MA (n = 109)	KA (n = 49)	MA (n = 59)	KA (n = 78)	Resurfacing^a^	Alignment^b^	Interaction
Sex					0.011	0.575	0.092
Female	77.1%	71.4%	52.5%	66.7%			
Male	22.9%	28.6%	47.5%	33.3%			
Age, mean (SD)	67.37 (8.75)	69.31 (10.66)	68.51 (6.96)	67.85 (8.88)	0.428	0.628	0.242
BMI, mean (SD)	31.17 (6.42)	30.48 (6.58)	29.94 (5.85)	30.53 (5.53)	0.969	0.556	0.398
ASA					0.170	0.696	0.580
1	4.6%	0%	1.7%	3.8%			
2	78.0%	85.7%	72.9%	74.4%			
3	17.4%	14.3%	25.4%	21.8%			
Smoking status					0.219	0.066	0.785
Current smoker	20.2%	12.2%	16.9%	15.4%			
Exsmoker	19.3%	16.3%	32.2%	21.8%			
Never smoked	60.6%	71.4%	50.8%	62.8%			

SD, standard deviation; ASA, American Society of Anesthesiologists.

a Significance level for resurfaced vs nonresurfaced patellae.

b Significance level for mechanical alignment vs kinematic alignment.

**Table 2:** Baseline radiographic, clinical and operative data for each of the subgroups of patients.

Baseline characteristic	Nonresurfaced		Resurfaced		*P* values		
	MA (n = 109)	KA (n = 49)	MA (n = 59)	KA (n = 78)	Resurfacing^b^	Alignment^c^	Interaction
Radiographical							
HKA angle, mean (SD)	4.36 (7.04)	5.47 (7.36)	5.24 (6.27)	3.58 (6.30)	0.141	0.129	0.099
Posterior tibial slope (°), mean (SD)	5.45 (3.46)	6.04 (3.37)	6.00 (3.45)	6.97 (4.01)	0.163	0.131	0.661
Patellar tilt (°), mean (SD)	6.05 (4.09)	6.78 (4.95)	6.29 (4.84)	6.82 (4.10)	0.958	0.500	0.862
Patellar height^a^, mean (SD)	0.89 (0.16)	0.88 (0.15)	0.87 (0.16)	0.91 (0.13)	0.194	0.096	0.125
Clinical							
WOMAC pain score, mean (SD)	34.82 (15.47)	36.36 (14.24)	38.02 (18.71)	39.19 (18.03)	0.362	0.748	0.935
WOMAC function score, mean (SD)	38.01 (21.12)	39.29 (14.39)	44.35 (20.24)	45.38 (19.26)	0.059	0.793	0.961
Operative							
Lateral release	17.0%	17.0%	12.1%	12.8%	0.299	0.920	0.924

MA, mechanical alignment; KA, kinematic alignment; HKA, hip-knee-ankle - positive value indicates varus deformity; SD, standard deviation.

a Caton-Deschamps index.

b Significance level for resurfaced vs nonresurfaced patellae.

c Significance level for mechanical alignment vs kinematic alignment.

### Patellar tilt

A greater patellar tilt was found at 1 year with kinematic (mean 7.31, SE = 0.48) than mechanical (mean 5.90, SE = 0.42) alignment (*P* = .028). Moreover, there were significant effects of resurfacing (*P* < .001) and sex (*P* = .011), which were qualified by a significant interaction (*P* = .041). Women with nonresurfaced patellae had a higher tilt (mean = 9.35, SE = 0.49) than with resurfaced (mean = 5.52, SE = 0.55) patellae (*P* < .001), whereas this difference was not found for men (nonresurfaced: mean = 6.38, SE = 0.81; resurfaced: mean = 5.19, SE = 0.66; *P* = .132). Other interactions were not significant. Descriptive statistics of patellar tilt at 6 weeks and 1 year are presented in Table 3.

**Table 3:** Patellar tilt at 6 wks and 1 y.

Patellar tilt (degrees)	Nonresurfaced		Resurfaced	
	MA	KA	MA	KA
At 6 wks				
Overall, mean (SD)	7.82 (4.68)	9.13 (5.60)	5.98 (4.35)	5.99 (4.18)
Women, mean (SD)	8.13 (4.88)	10.03 (5.59)	5.10 (3.46)	5.81 (4.14)
Men, mean (SD)	6.70 (3.78)	6.93 (5.17)	6.96 (5.06)	6.35 (4.31)
At 1 yr				
Overall, mean (SD)	7.63 (5.03)	9.43 (6.28)	4.49 (3.33)	6.22 (4.85)
Women, mean (SD)	7.89 (5.31)	10.80 (6.39)	4.84 (3.64)	6.19 (4.78)
Men, mean (SD)	6.76 (3.90)	6.00 (4.61)	4.11 (2.96)	6.27 (5.09)

*P* values (Patellar tilt at 1 y)						
Resurf	Align	Sex	Resurf × Align	Resurf × Sex	Align × Sex	Resurf × Align × Sex
<0.001	0.028	0.011	0.594	0.041	0.266	0.082

MA, mechanical alignment; KA, kinematic alignment; SD, standard deviation; Resurf, resurfacing; Align, alignment.

### WOMAC pain and function

As expected, WOMAC pain significantly improved from baseline (mean = 39.75) to 1 year (mean = 77.80, SE = 1.82), as did WOMAC function (baseline mean = 44.21, SE = 1.71; 1-year mean = 76.14, SE = 1.93). No effects of alignment technique on baseline-adjusted WOMAC pain (*P* = .902) and function (*P* = .921) were found at 1 year (Table 4). Moreover, 1-year patellar tilt did not correlate with WOMAC pain (r_s_ = −0.004; *P* = .954) or function (r_s_ = 0.016; *P* = .832) scores. WOMAC pain and function scores were available for 60% of included patients.

**Table 4:** Baseline-adjusted WOMAC pain and function at 1 y.

Outcome score	Nonresurfaced		Resurfaced		*P* values		
	MA (n = 61)	KA (n = 31)	MA (n = 31)	KA (n = 53)	Resurfacing^a^	Alignment^b^	Interaction
WOMAC pain score, mean (SD)	74.23 (22.77)	79.62 (16.89)	76.90 (17.69)	76.40 (17.19)	0.419	0.902	0.319
WOMAC function score, mean (SD)	74.14 (20.40)	78.59 (17.14)	73.04 (21.31)	73.52 (19.87)	0.236	0.921	0.529

MA, mechanical alignment; KA, kinematic alignment; SD, standard deviation.

a Significance level for resurfaced vs nonresurfaced patellae.

b Significance level for mechanical alignment vs kinematic alignment.

### Revisions and reoperations

Of the 295 included knees, 7 (2.4%) subsequently underwent revision surgery. Four of these were secondary patellar resurfacings, 1 exchange of polyethylene, 2 full revision TKA. Indications were PFJ problems (n = 2), aseptic loosening (n = 2), infection (n = 1), and persistent pain (n = 2). There was no difference in revision rates between alignment techniques: four (2.4%) MA and 3 (2.4%) KA knees were revised.

Fourteen knees (4.7%) underwent other reoperations. These were for manipulation under anesthesia for stiffness (n = 12), wound revision for dehiscence (n = 1), and washout of persistent hematoma (n = 1). There was no difference in reoperation rates between alignment techniques: 9 (5.4%) MA and five (3.9%) KA knees underwent reoperation (*P* = .783).

## Discussion

To our knowledge, this is the first clinical study in the literature reporting on the effects of KA on patellofemoral relationships in MS-TKA. It builds on previous work in the field with other types of TKA [23,24]. In this work, alignment technique did not affect WOMAC pain and function at 1-year postoperative for patients undergoing MS-TKA, with or without resurfacing of the patella. This is consistent with previous similar studies in cruciate-retaining TKA which demonstrated no difference in PROMs between MA and KA [23,[33], [34], [35]]. In our study, an overall increase in patellar tilt was found in KA TKA as compared with MA. Subgroup analysis by sex and resurfacing status found women with nonresurfaced patellae to be the only subgroup where this was the case, with a clinically insignificant mean difference of <4°. No difference in 1-year patellar tilt was observed in resurfaced patellae or in men. This finding of no clinically significant longer term patellar tilt is in line with Koh et al. They found no difference in postoperative patellar tilt between alignment techniques at 2-year follow-up in their study of an older TKA design which is no longer in common use [23]. Importantly, the literature disagrees regarding the threshold for a clinically significant patellar tilt [[19], [20], [21]]. It is therefore key that in this present study there was no correlation between 1-year patellar tilt and WOMAC pain or function score, suggesting when this difference in patellar tilt did occur, it was not clinically significant. This is consistent with work by Wen et al., where a greater patellar tilt was demonstrated for KA technique at 6 months postoperative, but there was no difference in Oxford Knee Score or ROM [24].

In the Koh et al. study, a limited range of femoral components, a patella-unfriendly implant and a polyradial femoral design were some of the contributory factors leading to concern around abnormal patella tracking and ongoing symptoms such as anterior knee pain. PFJ complications historically occurred in up to 50% of TKAs, but with the development of modern implants, rates are much less common [36]. Newer designs have attempted to tackle these issues by making changes which include reduced femoral component profile, improved anatomic trochlear groove, and medialized patellar shape. Still, such design improvements have been made in the setting of MA, and concerns remained that unrestricted KA might affect the position of the trochlea in some patients, with potentially worse outcome [37]. When longer term follow-up results are available, comparison of data from MA implants used for KA technique with KA-specific TKA would be extremely informative, especially regarding its effects on patellar tracking and PROMs.

There are some limitations to this study. This was a prospective register-based cohort, not a randomized control trial. Patients were recruited from a single center and operated on by a single surgeon using a single implant. This reduces the confounding factors introduced by comparing between different surgeons, institutions, or implants but can also affect the ability to generalize results. A further limitation is the exclusion of 34.9% of patients for missing data, with 93 of these known to have undergone MA, 45 to have undergone KA. Additionally, follow-up was to 1-year postoperative. Improvements in patient-reported outcome scores tend to plateau at this point [38,39], but a longer follow-up time period would be more informative with respect to implant survival. Phenotyping of the preoperative coronal deformities has not been done for the present study. This might be of interest to identify phenotype(s) at risk for worse outcome when using unrestricted KA with such implants designed for MA.

## Conclusions

The findings of this study suggest that although MS-TKA implants are designed for the MA philosophy, using the KA technique does not appear to adversely affect the biomechanics of the PFJ in a clinically significant manner. Patient-reported outcomes are also equivalent at 1 year postoperative. This provides reassurance that the advantages of the KA technique can be exploited in this type of implant.

## Conflicts of interest

S.W. King received institutional funding from B Braun to conduct a study related to wearable sensors in patients with TKA for an unconnected study and institutional funding from MEDACTA INTERNATIONAL S.A., Castel San Pietro, Switzerland for transport expenses to allow international collaboration. H. Pandit is a paid consultant for Zimmer Biomet, Medacta International, Allay Therapeutics, MATOrtho, Microport, Paradigm Pharmaceuticals, Teleflex, and Invibio; and owns stock or stock options in Allay Therapeutics. A. Lübbeke is an editorial board member of EFORT Open Reviews and is the president of the International Society of Arthroplasty registries. N. Silvestrini is an editorial board member of International Journal of Psychophysiology. H.H. Miozzari is an associate editor of the EFORT Open Review and a board member of Swiss Orthopaedics, Switzerland/member of the scientific advisory board SIRIS Hip & Knee.

For full disclosure statements refer to https://doi.org/10.1016/j.artd.2025.101750.

## CRediT authorship contribution statement

**Samuel W. King:** Writing – review & editing, Writing – original draft, Methodology, Investigation, Data curation, Conceptualization. **Nicolas Silvestrini:** Writing – review & editing, Formal analysis, Data curation. **Anne Lübbeke:** Writing – review & editing, Visualization, Supervision, Methodology, Investigation, Formal analysis, Data curation, Conceptualization. **Hemant Pandit:** Writing – review & editing, Visualization, Supervision, Project administration, Methodology, Funding acquisition, Conceptualization. **Hermes H. Miozzari:** Writing – review & editing, Supervision, Resources, Project administration, Methodology, Investigation, Conceptualization.

## Appendix A Supplementary data

Conflict of Interest Statement for Lübbeke

Conflict of Interest Statement for Miozzari

Conflict of Interest Statement for Pandit

Conflict of Interest Statement for Silvestrini

Conflict of Interest Statement for King

## References

[1] Culliford D., Maskell J., Judge A., Cooper C., Prieto-Alhambra D., Arden N.K. (2015). Future projections of total hip and knee arthroplasty in the UK: results from the UK Clinical Practice Research Datalink. Osteoarthritis Cartilage.

[2] Kurtz S., Ong K., Lau E., Mowat F., Halpern M. (2007). Projections of primary and revision hip and knee arthroplasty in the United States from 2005 to 2030. J Bone Joint Surg Am.

[3] Kurtz S.M., Ong K.L., Lau E., Widmer M., Maravic M., Gómez-Barrena E. (2011). International survey of primary and revision total knee replacement. Int Orthop.

[4] Baker P.N., van der Meulen J.H., Lewsey J., Gregg P.J. (2007). National joint registry for england and Wales. The role of pain and function in determining patient satisfaction after total knee replacement. Data from the national joint registry for England and Wales. J Bone Joint Surg Br.

[5] Ramkumar P.N., Harris J.D., Noble P.C. (2015). Patient-reported outcome measures after total knee arthroplasty. Bone Joint Res.

[6] Rivière C., Iranpour F., Auvinet E., Howell S., Vendittoli P.-A., Cobb J. (2017). Alignment options for total knee arthroplasty: a systematic review. Orthop Traumatol Surg Res.

[7] Whiteside A. (2013). The knee reconstruction, replacement, and revision.

[8] Hirschmann M.T., Karlsson J., Becker R. (2018). Hot topic: alignment in total knee arthroplasty-systematic versus more individualised alignment strategies. Knee Surg Sports Traumatol Arthrosc.

[9] Blakeney W., Clément J., Desmeules F., Hagemeister N., Rivière C., Vendittoli P.-A. (2019). Kinematic alignment in total knee arthroplasty better reproduces normal gait than mechanical alignment. Knee Surg Sports Traumatol Arthrosc.

[10] Howell S.M., Shelton T.J., Hull M.L. (2018). Implant survival and function ten years after kinematically aligned total knee arthroplasty. J Arthroplasty.

[11] Maheshwari A.V., Tsailas P.G., Ranawat A.S., Ranawat C.S. (2009). How to address the patella in revision total knee arthroplasty. Knee.

[12] Hull M.L., Simileysky A., Howell S.M. (2024). Differences in trochlear morphology of a new femoral component designed for kinematic alignment from a mechanical alignment design. Bioengineering.

[13] Rivière C., Harman C., Boughton O., Cobb J., Rivière C., Vendittoli P.-A. (2020). Personalized hip and knee joint replacement.

[14] Talbot S., Zordan R., Bennett K., Sasanelli F., Griffith A., Woodford N. (2023). Quadriceps tendon malalignment is an independent anatomical deformity which is the primary abnormality associated with lateral facet patellofemoral joint osteoarthritis. Knee Surg Sports Traumatol Arthrosc.

[15] Nisar S., Palan J., Rivière C., Emerton M., Pandit H. (2020). Kinematic alignment in total knee arthroplasty. EFORT Open Rev.

[16] Shatrov J., Battelier C., Sappey-Marinier E., Gunst S., Servien E., Lustig S. (2022). Functional alignment philosophy in total knee arthroplasty - rationale and technique for the varus morphotype using a CT based robotic platform and individualized planning. SICOT J.

[17] Ishikawa M., Kuriyama S., Ito H., Furu M., Nakamura S., Matsuda S. (2015). Kinematic alignment produces near-normal knee motion but increases contact stress after total knee arthroplasty: a case study on a single implant design. Knee.

[18] Nedopil A.J., Howell S.M., Hull M.L. (2017). What clinical characteristics and radiographic parameters are associated with patellofemoral instability after kinematically aligned total knee arthroplasty?. Int Orthop.

[19] Narkbunnam R., Electricwala A.J., Huddleston J.I., Maloney W.J., Goodman S.B., Amanatullah D.F. (2019). Suboptimal patellofemoral alignment is associated with poor clinical outcome scores after primary total knee arthroplasty. Arch Orthop Trauma Surg.

[20] Dahlmann S., Ziegeler K., Mau-Möller A., Mittelmeier W., Bergschmidt P. (2022). Patellar tracking in total knee arthroplasty—influence on clinical and functional outcome. Diagnostics.

[21] Bindelglass D.F., Cohen J.L., Dorr L.D. (1993). Patellar tilt and subluxation in total knee arthroplasty. Relationship to pain, fixation, and design. Clin Orthop Relat Res.

[22] van Houten A.H., Heesterbeek P.J.C., Wymenga A.B. (2016). Patella position is not a determinant for anterior knee pain 10 years after balanced gap total knee arthroplasty. Knee Surg Sports Traumatol Arthrosc.

[23] Koh D.T.S., Woo Y.L., Yew A.K.S., Yeo S.-J. (2021). Kinematic aligned femoral rotation leads to greater patella tilt but similar clinical outcomes when compared to traditional femoral component rotation in total knee arthroplasty. A propensity score matched study. Knee Surg Sports Traumatol Arthrosc.

[24] Wen L., Zhao X.X., Wang Z.W., Ma D.S., Zhang Q.X., Zhou L. (2022). [Comparative study on imaging and clinical results of patellofemoral joint with kinematic alignment and mechanical alignment in total knee arthroplasty]. Zhonghua Wai Ke Za Zhi.

[25] King S.W., Palan J., Pandit H. (2024). Medial stabilised total knee arthroplasty: definition and performance. J Orthop Exp Innov.

[26] Tawy G.F., Rowe P., Biant L. (2020). Advanced functional biomechanical analysis of medial rotation knee arthroplasty. Knee.

[27] Howell S.M. (2019). Calipered kinematically aligned total knee arthroplasty: an accurate technique that improves patient outcomes and implant survival. Orthopedics.

[28] Cooke D., Scudamore A., Li J., Wyss U., Bryant T., Costigan P. (1997). Axial lower-limb alignment: comparison of knee geometry in normal volunteers and osteoarthritis patients. Osteoarthritis Cartilage.

[29] Gomes L.S., Bechtold J.E., Gustilo R.B. (1988). Patellar prosthesis positioning in total knee arthroplasty. A roentgenographic study. Clin Orthop Relat Res.

[30] Brazier J., Migaud H., Gougeon F., Cotten A., Fontaine C., Duquennoy A. (1996). [Evaluation of methods for radiographic measurement of the tibial slope. A study of 83 healthy knees]. Rev Chir Orthop Reparatrice Appar Mot.

[31] Caton J., Deschamps G., Chambat P., Lerat J.L., Dejour H. (1982). [Patella infera. Apropos of 128 cases]. Rev Chir Orthop Reparatrice Appar Mot.

[32] Konrads C., Grosse L.C., Ahmad S.S., Springer F., Schreiner A.J., Schmidutz F. (2021). Reliability of a Caton-Deschamps-derived patella height index for knee arthroplasty. Int Orthop.

[33] Howell S.M., Howell S.J., Kuznik K.T., Cohen J., Hull M.L. (2013). Does a kinematically aligned total knee arthroplasty restore function without failure regardless of alignment category?. Clin Orthop Relat Res.

[34] Young S.W., Walker M.L., Bayan A., Briant-Evans T., Pavlou P., Farrington B. (2017). The Chitranjan S. Ranawat award : No difference in 2-year functional outcomes using kinematic versus mechanical alignment in TKA: a randomized controlled clinical trial. Clin Orthop Relat Res.

[35] Waterson H.B., Clement N.D., Eyres K.S., Mandalia V.I., Toms A.D. (2016). The early outcome of kinematic versus mechanical alignment in total knee arthroplasty: a prospective randomised control trial. Bone Joint J.

[36] Assiotis A., To K., Morgan-Jones R., Pengas I.P., Khan W. (2019). Patellar complications following total knee arthroplasty: a review of the current literature. Eur J Orthop Surg Traumatol.

[37] Howell S.M., Sappey-Marinier E., Niesen A.E., Nedopil A.J., Hull M.L. (2023). Better forgotten joint scores when the angle of the prosthetic trochlea is lateral to the quadriceps vector in kinematically aligned total knee arthroplasty. Knee Surg Sports Traumatol Arthrosc.

[38] Wylde V., Penfold C., Rose A., Blom A.W. (2019). Variability in long-term pain and function trajectories after total knee replacement: a cohort study. Orthop Traumatol Surg Res.

[39] Clement N.D., Afzal I., Demetriou C., Deehan D.J., Field R.E., Kader D. (2020). There is no clinically important difference in the Oxford knee scores between one and two years after total knee arthroplasty: the one-year score could be used as the benchmark timepoint to assess outcome. Knee.

