# Supplementary material for: Does Alignment Technique in Medially Stabilized Total Knee Arthroplasty Affect the Patellofemoral Joint Biomechanics and Patient-reported Outcomes at 1 Year? A Prospective Registry-based Cohort Study
Source: Arthroplast Today. 2025 Jun 26;34:101750. doi: 10.1016/j.artd.2025.101750 (PMC12241387; doi:10.1016/j.artd.2025.101750)
Supplement: Conflict of Interest Statement for Silvestrini [file mmc4.pdf]

INDIVIDUAL CONFLICT OF INTEREST STATEMENT

American Association of Hip and Knee Surgeons

(Adopted from the American Academy of Orthopaedic Surgeons disclosure statement)

The following form **must be filled out completely and submitted by each author (example, 6 authors, 6 forms). All items require a response. If there is no relevant disclosure for a given item, enter "*None.*"**

Manuscript Title: Does Alignment Technique in Medially Stabilised Total Knee Arthroplasty Affect the Patello-Femoral Joint Biomechanics and Patient-Reported Outcomes at One Year? A Prospective Registry-Based Cohort Study.

1. Royalties from a company or supplier (The following conflicts were disclosed)

None

2. Speakers bureau/paid presentations for a company or supplier (The following conflicts were disclosed)

None

3A. Paid employee for a company or supplier (The following conflicts were disclosed)

None

3B. Paid consultant for a company or supplier (The following conflicts were disclosed)

None

3C. Unpaid consultants for a company or supplier (The following conflicts were disclosed)

None

4. Stock or stock options in a company or supplier (The following conflicts were disclosed)

None

5. Research support from a company or supplier as a Principal Investigator (The following conflicts were disclosed)

None

6. Other financial or material support from a company or supplier (The following conflicts were disclosed)

None

7. Royalties, financial or material support from publishers (The following conflicts were disclosed)

None

8. Medical/Orthopaedic publications editorial/governing board (The following conflicts were disclosed)

None

9. Board member/committee appointments for a society (The following conflicts were disclosed)

Editorial board member of the International Journal of Psychophysiology

Each author must sign AND print or type his/her name, date and submit a separate form

In addition, one BLINDED Conflict of Interest form (no author names used) should be submitted per manuscript with all author disclosures.

Nicolas Silvestrini	Silvestrini	03/12/2024
Author Name (Print or Type)	Author Signature	Date
