# Supplementary material for: Does Alignment Technique in Medially Stabilized Total Knee Arthroplasty Affect the Patellofemoral Joint Biomechanics and Patient-reported Outcomes at 1 Year? A Prospective Registry-based Cohort Study
Source: Arthroplast Today. 2025 Jun 26;34:101750. doi: 10.1016/j.artd.2025.101750 (PMC12241387; doi:10.1016/j.artd.2025.101750)
Supplement: Conflict of Interest Statement for King [file mmc5.docx]

# INDIVIDUAL CONFLICT OF INTEREST STATEMENT

***American Association of Hip and Knee Surgeons***

(Adopted from the American Academy of Orthopaedic Surgeons disclosure statement)

The following form **must be filled out completely and submitted by each author (example, 6 authors, 6 forms).**

**All items require a response. If there is no relevant disclosure for a given item, enter "*None*.”**

**Manuscript Title** Does Alignment Technique in Medially Stabilised Total Knee Arthroplasty Affect the Patello-Femoral Joint Biomechanics and Patient-Reported Outcomes at One Year? A Prospective Registry-Based Cohort Study

1. Royalties from a company or supplier (The following conflicts were disclosed)

None

2. Speakers bureau/paid presentations for a company or supplier (The following conflicts were disclosed)

None

3A. Paid employee for a company or supplier (The following conflicts were disclosed)

None

3B. Paid consultant for a company or supplier (The following conflicts were disclosed)

None

3C. Unpaid consultants for a company or supplier (The following conflicts were disclosed)

None

4. Stock or stock options in a company or supplier (The following conflicts were disclosed)

None

5. Research support from a company or supplier as a Principal Investigator (The following conflicts were disclosed)

Institutional funding from B Braun to conduct a study related to wearable sensors in patients with TKA for an unconnected study

Institutional funding from MEDACTA INTERNATIONAL S.A., Castel San Pietro, Switzerland for transport expenses to allow international collaboration

6. Other financial or material support from a company or supplier (The following conflicts were disclosed)

None

7. Royalties, financial or material support from publishers (The following conflicts were disclosed)

None

8. Medical/Orthopaedic publications editorial/governing board (The following conflicts were disclosed)

None

9. Board member/committee appointments for a society (The following conflicts were disclosed)

None

**Each author must sign AND print or type his/her name, date and submit a separate form**

In addition, one BLINDED Conflict of Interest form (no author names used) should be submitted per manuscript with all author disclosures.

Samuel King
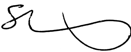
 03/12/2024

Author Name (Print or Type) Author Signature Date
